# Evaluation of Performance, Nitrogen Metabolism and Tissue Composition in Barrows Fed an n-3 PUFA-Rich Diet

**DOI:** 10.3390/ani9050234

**Published:** 2019-05-13

**Authors:** Mihaela Hăbeanu, Nicoleta Aurelia Lefter, Anca Gheorghe, Arabela Untea, Mariana Ropotă, Daniela-Mihaela Grigore, Iulia Varzaru, Smaranda Mariana Toma

**Affiliations:** National Research Development Institute for Animal Biology and Nutrition, IBNA Balotesti, Calea Bucuresti nr. 1, Ilfov 077015, Romania; nicoleta.ciuca@ibna.ro (N.A.L.); anca.gheorghe@ibna.ro (A.G.); arabelapatrichi2002@yahoo.com (A.U.); m.ropota@yahoo.com (M.R.); daniela.grigore@ibna.ro (D.-M.G.); iulia_maros@yahoo.com (I.V.); smaranda.pop@ibna.ro (S.M.T.)

**Keywords:** nitrogen, balance, fatty acids, amino acids, plasma, tissue, pigs

## Abstract

**Simple Summary:**

The metabolic response of pigs to nutritional treatment was highlighted in a variety of ways. Since the literature related to the effects of an n-3-rich diet on product quality have shown a number of health advantages, one of the next steps could be the consideration of the relation between an n-3 polyunsaturated fatty acid (PUFA)-rich diet and nutrient metabolism with a focus on nitrogen as an important indicator of protein synthesis and degradation. It is important to understand how unconventional diets affect the environment with respect to consumer demand regarding the quality of products and the health status of animals. Therefore, novel compound feed formulas to improve feeding efficiency by decreasing the n-6:n-3 fatty acid ratio are needed. However, it is assumed that by changing the fatty acid profile, certain plasma or tissue parameters could be directly affected as well. In our study, we modified the n-6:n-3 ratio in different barrow tissues by the dietary addition of extruded linseed associated with walnut meal employing a mixture of 50:50 (*wt/wt*) n-3 fatty acids as dietary enrichment. This paper also gives information about the influence of extruded linseed/walnut meal mixture on performance and nitrogen metabolism in barrows.

**Abstract:**

We investigated changes in nitrogen metabolism and chemical, fatty acid (FA) and amino acid (AA) composition in tissues (longissimus dorsi (LD) and semitendinosus (ST) muscles, heart, spleen, liver and cecum) following the dietary addition of extruded linseed and walnut meal (50:50 mix). Plasma creatinine and urea nitrogen were determined as well. Two trials were designed using barrows (five replicates) allotted in two groups [(control, (SM) and experimental, (LEW))] in metabolic cages. The experimental diet rich in n-3 FA led to a significant increase of retained nitrogen (>8.09% in the LEW group). The biological value of feed protein was 14.8% higher in the LEW group than in the SM group. LD muscles from the SM group and liver from the LEW group had greater nitrogen contents, whereas the heart and spleen had lower concentrations of nitrogen in the LEW group. Diet had a pronounced effect on n-3 FA, particularly on α-linolenic fatty acid (ALA) (*p* < 0.0001). The highest levels of ALA were recorded in the cecum (>6.06 times in LEW) and heart (>5.44 times in LEW). The highest level of lysine was noticed in the LD muscle (>2.1% in SM). An n-3-rich diet significantly reduced the amount of nitrogen excreted; greater than 40% nitrogen was retained, thus improving the meat composition.

## 1. Introduction

The trend of an increasing consumption of animal-origin food products, as well pork per capita, has continued, thus requiring an increase in livestock production. Growing concentrations of animals lead to increased output. However, the consequences of this have been reflected in an increased food safety risk, as well as effects on health and the environment with profound implications in our lives. Thus, safety measures are required, with a focus on the livestock sector. Over time, nutritionists have highlighted that a number of feedstuffs or byproducts from the industry sector can beneficially modulate animal product quality and subsequently have an impact on health status. Previous studies have led to the assumption that, while certain health markers or lipid structures are changed, the digestibility of some nutrients may be altered as well. A major concern is feeding efficiency, as an important indicator of the sustainability of pig production [1,2]. The biological mechanism of this indicator is not well elucidated. It has been measured by gain:feed or residual feed intake [3,4]. For many years, gain:feed has been known as an indicator for profitability, being related to body weight as well as gain and feed composition. We assert that a measure of feeding efficiency consists of decreasing inputs based on a higher quality of feedstuff. This leads to a higher metabolic efficiency in using nutrients. Moreover, the level of nutrients excreted decreases. It is known that nitrogen losses affect the environment and 70% of NH_3_ released into the environment originates from the livestock sector [5]. The most important portion of nitrogen in animal metabolism is that derived from protein. It gives us an indication of protein synthesis and degradation. About 5% to 30% of nitrogen is converted to food for human diets and the rest is excreted by animals. Furthermore, an excess of nitrogen will affect attempts to meet Sustainable Development Goals.

On the other hand, in choosing vegetable resources as well as byproducts from the industry sector, we aim to improve not only the livestock productivity and alleviate the environmental burden, but also improve the quality of the products in order to meet consumer requirements. The goal was to increase the consumption of saturated fat by increasing feed use and feed quality. Dugan et al. [6] specified that, due to the proven positive effects of n-6:n-3 fatty acids (FA) in comparison to saturated FA, this knowledge has increased the tendency to select healthful resources for animal feeding. The ingredients selected manipulate the ratio of n-6:n-3 PUFA (polyunsaturated FA) toward a level beneficial for health.

Extruded linseed and walnut meal are both useful resources if we consider them as dietary additions to enrich the levels of n-3 FA. Moreover, these resources have valuable amino acid (AA) compositions. Extruded linseed has a higher level of methionine + cysteine (Met + Cys), whereas walnut meal is richer in Lysine (Lys). In order to improve n-3 FA level in the diet we used a mixed between these two resources (50:50 *wt/wt* mix). Any increase of production also implies an increase of cereal consumption, which depletes the amount available for human consumption. Usually corn is used in a classical diet, but in our study, we added millet as a cereal with excellent nutritional value. It is known the heart, liver and spleen are key organs in relation to health status and have a nutrient-dense composition, but little is known about their detailed chemical content in FA and AA. In the present paper, the chemical FA and AA composition were assessed in different tissue due to their role in lipoprotein metabolism and their importance as edible organs. Our information regarding lipids structure in edible organs modified by diet, could help consumers meet health recommendations if we take into consideration the n-3 PUFA potential therapeutic roles in intestinal inflammation in pigs, in the regulation of hepatic lipid metabolism, adipose tissue function and inflammation, to help reduce the risk of heart disease and also to promote healthy skin. 

This study is part of a nutritional program that aims to increase efficiency in the use of nutrients from n-3-rich compound feed formula and to determine its relation to health markers and product quality. The current research allowed us to evaluate changes in performance, nitrogen metabolism and composition of different tissues (longissimus dorsi (LD) and semitendinosus (ST) muscles, heart, spleen, liver and cecum) by using a mix (50:50 wt/wt) between two sources rich in n-3 FA, especially in α-linolenic FA (ALA).

## 2. Materials and Methods

### 2.1. Experimental Design and Diets

After ethics committee approval (1493/12.03.2018), two trials were carried out on barrows at National Research Development Institute for Animal Biology and Nutrition (INCDBNA Balotesti) according to Romania Law 43/2014 for animal care. Two diets were based on corn, millet and soybean meal in the SM group and 60 g/Kg, (50:50, *wt/wt*) mix of extruded linseed and walnut meal (EL:WM) replaced 14.2% SM in the experimental group (linseed extruded and walnut meal, LEW) (Table 1). 

Diets were formulated to contain 9.7 g·kg^−1^ Lys (as-fed basis), 8.0–8.1 g·kg^−1^ standardized ileal digestible (SID) of amino acids SID Lys, 6.0–6.2 g·kg^−1^ Met + Cys, 5.0–5.3 g·kg^−1^ SID Met + Cys, respectively. The limiting amino acids for pigs (Lys, Met and Cys) had similar levels in the diet by the supplementary addition of synthetic L-Lysine HCl and DL-methionine. 

The EL:WM 50:50 *wt/wt* mix was ground and processed separately. Extruded linseed had 50.57% ALA (% of total fatty acid methyl esther, FAME) and 20.29% LA while walnut meal had 10.94% ALA and a higher concentration of linoleic acid (LA) (60.79%). The addition of the EL:WM mix led to a 7.81% decrease of the n-6:n-3 ratio in the diet.

Ten crossbred barrows Topigs ((♀ Large White × Hybrid (Large White × Pietrain) × ♂ Talent, (mainly Duroc)), 81 ± 3 days old, 31.5 (SD 1.90) kg initial weight, were randomly assigned for three weeks (first week for accommodation) to two groups (SM and LEW), each with five replicates. The animals’ weight was recorded at the start and the end of experiment. Barrows were housed in individual metabolic cages (1.2 × 1.5 × 1 m). The temperature during the experiment was 22 ± 1 °C. Feed was given ad libitum twice a day (08.00 h and 15.00 h) and water was supplied after the first meal. 

### 2.2. Trial 1: Nitrogen Balance, Plasma Creatinine and Urea Concentration 

The first trial aimed to assess nitrogen digestibility and nitrogen retention (NR), biological value of feed protein (BVFP), net protein utilization (NPU), coefficient of metabolizability (CAM) and plasma nitrogen, creatinine and urea. 

The nitrogen balance experiment was carried out in an atmosphere-controlled room, equipped with steel individual metabolic cages. After 7 days of adaptation, two balance periods followed during which fecal matter and urine were quantitatively collected, at 08.00–08.30 h. Samples obtained were stored at 5 °C. After the balance period, feces collected from each animal were pooled, homogenized and 10% of samples were frozen at −18 °C for analyses.

Urine volume was recorded daily and 10% aliquots were preserved at −18 °C for analyses. In order to reduce pH and conserve nitrogen, we used H_2_SO_4_ 25% concentration in each urine container. 

Pig feces samples of 0.4 g were weighed with an accuracy of ±2 × 10^‒4^ g. Each sample was processed as described in [8]. A blank digest was carried out in the same way. A semiautomatic Kjeldahl method (Kjeltec Auto 1030 Analyzer, Hillerod, Denmark) was used to determine the nitrogen content in the excretions. The samples were digested using H_2_SO_4_ in the presence of catalyzers, followed by distillation and titration. Class A glassware was used for transvasation, dilution and storage. All reagents used were supplied by Merck (Darmstadt, Germany). For calibration stock solutions traceable to standard reference material (SRM) from the National Institute of Standards and Technology (NIST) were used for calibration. Nitrogen digestibility as well as balanced or retained nitrogen (NR) were determined by measuring nitrogen intake (dry matter (DM) basis) and nitrogen excretion. Nitrogen absorption (NAB), BVFP and NPU were calculated according to [9]. The coefficient of total tract apparent (CTTAD) and CAM were calculated using equations as shown in [10]. 

At the end of the experiment, blood samples were collected by jugular venipuncture in heparin tubes (2 samples/animal), and then centrifuged (3000 rpm for 15 min) for plasma separation. A chemistry analyzer (Spotchem EZ SP-4430) was used in order to assess nitrogen, creatinine and urea nitrogen (PUN). 

### 2.3. Trial 2: Performance and Tissue Chemical Composition

Trial 2 aimed to determine performance, tissue levels of n-6 and n-3 FA and tissue levels of limiting AA (Lys, Met and Cys). 

Barrow weights were recorded at the beginning of the first balance period and at the end of the experiment. At the end of balance experiment, the animals were slaughtered following the procedure specified in legislation. Around 200 g samples of muscle (LD and ST) and organ (spleen, heart, liver and cecum) were taken in order to determine chemical, FA and AA compositions. The tissue samples were ground using an IKA^®^ A 11 basic analytical mill (Werke GmbH & Co. KG, 79219 Staufen Germany) and liquid nitrogen. 

The gross chemical composition of the feed and tissue were evaluated by methods approved by Commission Regulation (EC) no. 152 (OJEU, 2009). 

HPLC Surveyor Plus Thermo Electron equipment (Waltham, MA, USA) and a HyperSil BDS C18 column (Thermo Electron, Waltham, MA, USA) with dimensions of 250 mm × 4.6 mm × 5 μm were used to determine the AA profiles of the tissue samples. Samples were prepared as described previously [11]. 

A Perkin Elmer-Clarus 500 gas chromatograph (Waltham, MA, USA), fitted with a flame ionization detector (FID) and capillary separation column with high polar stationary phase Agilent J&WGC Columns (Thermo-Electron Corporation, Waltham, MA, USA), DB-23 with dimensions of 60 m × 0.250 mm × 0.25 μm, was used for FA determination (method described in [12]).

### 2.4. Calculation and Statistical Analysis 

Based on experimental data, NAB, NR, total nitrogen output (TNO), BVFP, NPU, CTTAD and CAM were calculated according to previously developed equations [9,10,13]. Nitrogen digestibility, and balanced or retained nitrogen (NR) were determined by measuring nitrogen intake (DM basis) and nitrogen excretion. TNO was calculated as the difference between nitrogen intake and fecal nitrogen. BVFP was calculated as the ratio of NR to NAB. NPU was calculated as the ratio of NR to NI. CTTAD and CAM were calculated using the following equations: CAM = ((N intake − N fecal output − N urinary output)/N intake); CTTAD = ((N intake – fecal N output)/N intake).

Response data, expressed as the mean and standard error of the mean (SEM), were analyzed by a general linear model multivariate procedure using Software Statistical Package SPSS, version (2011) with diet as the main factor. We considered the response to the treatment as the dependent variable, the diet and/or tissue as fixed factors. The significance of specific effects of diet, tissue, as well as diet × tissue in trial 2 was determined. The Pearson’s correlation was used to evaluate the relation between certain parameters. Diﬀerences were signiﬁcant if *p* < 0.05, highly significant when *p* < 0.001 or *p* < 0.0001 and a trend was considered if 0.05 ≤ *p* < 0.10. Standard ileal digestibility calculated values were based on theoretical data from [7] and analyzed amino acid concentrations except for walnut meal. Stepwise regression analysis–SPSS software was used to determine SID AA for walnut meal.

## 3. Results

### 3.1. Trial 1: Nitrogen Balance, Plasma Creatinine and Urea Concentration 

No health problems were noticed during the metabolic experiment. According to the data shown in Table 2, there was no significant effect of the treatment on feed intake, dry matter (DM) or nitrogen intake.

Although the nitrogen intake was similar between treatments, a decrease of 14.66% (*p* < 0.05) was noticed in TNO in the LEW group compared to the SM group. The dietary addition of extruded linseed and walnut meal led to a high reduction of the nitrogen level in urine (1.31 times, *p* < 0.01) and in calculated NAB (1.04 times, *p* < 0.05). Calculated BVFP was 14.8% higher (*p* < 0.05) in the LEW group compared to the SM group. In addition, an increase of NPU (*p* > 0.05) in the LEW group compared to the SM group was not obtained. Dietary treatments did not affect CTTAD or CAM (*p* > 0.05).

As expected, a greater significant positive correlation was noticed between NR and BVFP values (0.92, *p* < 0.0001), as well as between NR and NPU (0.999, *p* < 0.001). Regarding the correlation between NR and PUN, we noticed a significant correlation (0.54, *p* < 0.001). The plasma creatinine concentration decreased 11.36% in the LEW group compared to the SM group (*p* < 0.05).

### 3.2. Trial 2: Performance and Tissue Composition 

#### 3.2.1. Growth Performance

Extruded linseed used in this study had 18.36% protein content and 21.18% ether extract (EE), whereas walnut meal had a higher level of protein (36.53%) than EE (7.42%). The diets had similar content in metabolizable energy, protein, fat and amino acids. As shown in Table 3, no significant difference was noticed on growth performance. 

Barrows fed with EL:WM had 3.63% higher average daily gain (ADG) than barrows fed with SM. A decrease by 5.22% of the gain:feed ratio was observed in the LEW group. A negative correlation was noticed between plasma creatinine and body weight gain (BWG) (r = −0.44, *p* < 0.001).

#### 3.2.2. Tissue Chemical Composition 

The data concerning the chemical composition of tissues are shown in Table 4. 

LD muscle had a nitrogen content lower in the LEW group (*p* > 0.05) compared to the SM group. In contrast, in ST muscle, heart, spleen and liver, we noticed an increase in nitrogen content. No diet × tissue interaction was observed (Table 4). LD is known to be more oxidative than the ST muscle [12]; thus, we found LD to contain <5.5% fat in the LEW group vs. the SM group. Nonetheless, a higher fat level was found in ST muscle (>11.06% in the LEW group) and spleen (>12.99% in the LEW group).

In our study, we recorded nitrogen values similar to those found by the authors of [14] in the heart, spleen and liver. We found a negative correlation (*p* > 0.05) with the fat content of these tissues. 

#### 3.2.3. Distribution of Fatty Acids and Amino Acids

Changes in the distribution of FA in different tissues caused by diet enrichment in n-3 PUFA are presented in Table 5. 

As expected, diet had a more pronounced effect on the concentration of n-3 FA, particularly on ALA (*p* < 0.0001). The higher level of ALA was recorded in cecum (>6.06 times in the LEW group compared to the SM group), followed by heart (>5.44 times in the LEW group compared to the SM group) and liver (>4.89 times in the LEW group compared to the SM group). Although LD and ST muscles are distinguishable by the metabolic activity of their fiber (LD has more oxidative and ST has more glycolytic activity [12]), the ALA concentrations were similar in both. A greater decrease of the n-6:n-3 ratio was noticed in the LEW group, irrespective of the tissue (*p* < 0.01). Thus, a lower n-6:n-3 ratio was noticed in the spleen, liver and cecum (*p* < 0.05). A significant diet × tissue interaction was noticed in LCFA (long chain FA; docosapentaenoic acid (DPA) and docosahexaenoic acid (DHA), ⅀n-3 and n:6-n-3 ratio. A tendency regarding the additive effects of diet and tissue were observed for ALA. We found a negative correlation between dietary n-6:n-3 and tissue ALA (r = –0.65, *p* < 0.01), while dietary ALA was positively and significantly correlated with tissue ALA concentration (r = 0.65, Table 6). A correlation tendency was observed between DPA and nitrogen level in tissue (*p* < 0.10). A negative correlation was found between ALA in tissue and TNO (r = −0.57, *p* < 0.01) and a positive correlation was found between dietary n-6:n-3 and tissue n-6:n-3 (r = 0.51, *p* < 0.01). A positive relationship was also registered between n-6:n-3 ratio in diet or tissue and urinary nitrogen output (r = 0.78, *p* < 0.001).

Each body tissue has a specific concentration of amino acids. In Table 7, the limiting amino acids found in samples of tissue (LD, ST, heart, spleen and liver) are presented. Lysine is the main limiting amino acid for pigs, followed by methionine, cysteine, threonine and tryptophan. 

We identified only traces of tryptophan in our samples. The highest level of Lys was found in the LD muscle (>2.1% in the SM group) and heart (43.71% higher in the LEW group). Except in the heart, the dietary addition of EL:WM increased the concentration of Met (*p* < 0.01). Regarding Cys, the same tendency was observed except in the spleen. A negative positive correlation was found between nitrogen intake and Cys + Met (*p* < 0.005). A significant diet × tissue interaction was noticed for Cys (*p* < 0.05). Met + Cys were negatively correlated with TNO (r = −0.57, *p* < 0.01).

## 4. Discussion

Animals use dietary protein nitrogen to replace digestive enzymes and different compounds that are degraded and lost. It has been acknowledged that we should decrease the diet pollutant component that is excreted as much as possible. Many previous studies have highlighted that nitrogen output can be decreased, particularly through nutritional approaches [9,10,15,16]. Previous research has focused on the digestibility of nutrients supplied by protein and fiber-rich feedstuff. However, we noted a lack of information regarding the availability of nutrients from n-3-rich diets. In this study, we evaluated changes in nitrogen output, absorbed and retained nitrogen, and tissue composition as the effects of a mixture between EL:WM characterized by a higher content in n-3 PUFA. The relations between nitrogen digestibility and plasma parameters such as PUN and creatinine were determined as well. Our diet had a higher n-3 FA level as compared to the traditional diet, obtained by the addition of extruded linseed in association with walnut meal. An increasing emphasis is being place on pollution due to nitrogen from the livestock sector. Our question is how to feed piglets without negatively affecting the environment while also meeting consumer demands regarding the quality of products and the health status of the animals. Extruded linseed (n-3-rich ingredient) in association with walnut meal (n-6-rich byproduct) increased the n-3 FA composition in the diet 6.83 times and increased the ALA composition 8.11 times compared to the SM diet. The n-3:n-6 PUFA ratio in the LEW diet was 2.56. This was established as a ratio close to 1 which is required for the prevention of certain diseases [17,18,19]. 

The results obtained in this work suggest that although nitrogen intake was similar between groups, TNO was significantly reduced (<14.66%) by the dietary addition of EL:WM. Known as a biological marker for protein intake [20], in our study urine nitrogen excretion was found to be positively correlated with nitrogen intake (*p* < 0.01). The decrease of urinary nitrogen output (*p* < 0.01) in the LEW group had a significant effect on TNO. A positive nitrogen balance was noticed, indicating that the nitrogen intake exceeded the nitrogen lost. The NR value was slightly higher in the LEW group, and was positively correlated with BVFT and NPU. The current results are close to those obtained in [21] using *Vachellia tortilis* leaf meal, although the percent of nitrogen retained was higher in our research (>46% compared to 42%), probably due to the lower fecal nitrogen output in our case. In a previous study [22], two levels of protein and inulin in the diet were used, and a similar fecal nitrogen level was found, while the urinary nitrogen resulted as higher than the level obtained in our study. 

It is known that blood parameters serve as markers that give some information about animal health status (physiological, nutritional, pathological changes) as well as the quality of feed. Following the catabolism of AA, urea is the principal nitrogen end product used as an index of animal ability to retain dietary nitrogen and an indicator of feed efficiency [23,24]. In our study, PUN and creatinine were within the physiological interval (8.2–25 mg/dL for urea, 0.8–2.3 mg/dL for creatinine, [25,26,27] which suggests that there is no negative influence of the diets. Despite the similar levels of AA in the diet, the PUN concentration increased 5.8% in the LEW group, leading to an increase of urea excretion as result of AA catabolism [9]. Like urea, creatinine is a non-protein nitrogen. The creatinine concentration was markedly lower in the LEW group compared to the SM group. As expected, when PUN increased creatinine decreased, while the PUN:creatinine ratio increased significantly. Previous studies have shown that an increase in the PUN concentration improves protein accretion [28].

A marked influence was established on n-3 FA, especially ALA, by the dietary addition of n-3-rich supplements at the tissue level. The most pronounced effect was noticed in the heart (>6.88 times), followed by the cecum (>6.06 times) and liver (>4.89 times). It was specified previously that DPA is an intermediate for DHA synthesis from EPA [6,12]. This long chain (LC) FA exhibited a greater concentration than other LC FA. Although in the LEW group DPA was lower, possibly because a large part was converted to DHA (*p* < 0.01), only a trace of EPA was found. As remarked [29], dietary linseed determines a rapid response in animal organisms. However, the response also depends on quantity and time. The diet with n-3 enrichment (by EL:WM addition) in our study had a positive effect on total n-3 FA and on the n-6:n-3 ratio (*p* < 0.01). Except in the case of ALA, tissue type had a pronounced effect on the essential n-3 and n-6 FA. 

The carbon skeletons of essential AA are not synthesized in vivo [29]. However, given their importance for animal health, we supplemented diets in order to ensure their specific requirements. As mentioned above, the first limiting AA in swine nutrition is Lys, which is a ketogenic AA. Lys concentration increased in tissue of the LEW group by 10.9%. It is established that Lys decreases the nitrogen excretion in urine in association with TNO and maintains performance [24]. Although the diets had similar Lys contents, the LEW diet increased the concentration of this AA in various tissues (43% in heart, 9% in spleen and 4% in liver, *p* > 0.05). On the contrary, in LD and ST muscle the Lys level was slightly reduced (*p* > 0.05). Sulphur-containing AA concentration, Met and Cys all showed highly significant differences between the LEW diet and the SM diet. The greater proportion was observed in the LD muscle and heart. Met + Cys were negatively correlated with TNO (r = −0.57, *p* < 0.01). 

## 5. Conclusions

Nitrogen is vital for life and a very important nutrient. Overall, the results show that n-3 FA enrichment diet had a significant impact on nitrogen metabolism. The addition of LE:WM mixture reduced nitrogen excreted and net protein utilization. More than 40% of nitrogen was retained which led to a higher efficiency of nitrogen utilization. The dietary addition of extruded linseed associated with walnut meal favored deposition in the tissue of n-3 PUFA, especially ALA of which their health effects are well known.

## Figures and Tables

**Table 1:** Structure and nutritional value of experimental diets.

Ingredients g/kg (as-Fed Basis)	Control (SM Diet) ^a^	Experimental (LEW Diet) ^b^
Ground corn	380.4	343.0
Millet	250	250
Rice flour	170	170
Extruded linseed:walnut meal, 50:50 (*wt/wt*) mix	0	60
Soybean meal (44%)	160	140
Soybean oil	5	2
DL-methionine	0.3	0.7
L-Lysine HCl	2.7	2.7
Carbonate calcium	14.5	14.5
Phytase	0.1	0.1
Monocalcium phosphate	5	5
Salt	1	1
Premix choline	1	1
Vitamin/mineral premix ^c^	10	10
Nutritional value g/kg (as-fed basis)		
Metabolizable energy (EM, MJ/kg)	12.81	12.80
Crude protein	160	164.2
Lys ^d^	9.7	9.7
SID Lys ^4^	8.0	8.1
Met + Cys ^d^	6.0	6.2
SID Met + Cys ^4^	5.0	5.3
Calcium	8.0	8.0
Total Phosphorus	5.9	5.9
Fatty acids composition (% FAME)		
ALA ^d^	1.88	15.25
LA ^d^	50.73	39.68
⅀ n-3	2.28	15.58
⅀ n-6	51.4	40.0
n-6:n-3 PUFA	22.54	2.56

^a,b^ Soybean meal diet; extruded linseed + walnut meal diet. ^c^ Vitamin/mineral premix provided per kg diet: 6000 IU vitamin A; 800 IU vitamin D3; 20 IU vitamin E; 1 mg vitamin K3; 1 mg vitamin B1; 3.04 mg vitamin B2; 10 mg vitamin B3; 6.3 mg vitamin B5; 1.5 mg vitamin B6; 0.03 mg vitamin B7; 0.3 mg vitamin B9; 0.02 mg vitamin B12; 30 mg Mn; 80 mg Fe; 25 mg Cu; 100 mg Zn; 0.22 mg I; 0.22 mg Se; 0.3 mg Co; 60 mg antioxidant. ^d^ Lys = lysine; Met = methionine; Cys = cysteine; ^4^ SID: standard ileal digestibility calculated values based on theoretical data from [7] and analyzed amino acid concentrations except for walnut meal. Stepwise regression analysis–SPSS software was used to determine amino acid (AA) SID for walnut meal. ALA = α-linolenic acid; LA = linoleic acid; PUFA = polyunsaturated fatty acid.

**Table 2:** Effect of dietary extruded linseed:walnut meal 50:50 (*wt/wt)* mix on feed intake, nitrogen intake, nitrogen output, retained and absorbed nitrogen, digestibility, plasma urea nitrogen and creatinine concentration in grower barrows pigs.

ITEMS (g/day) **	SM Diet	LEW Diet	SEM	*p* Value *
Feed intake	2780	2770	0.05	0.95
Dry matter (DM) intake	2460	2450	0.04	0.95
Nitrogen intake	70.76	72.91	0.45	0.46
Fecal DM	251.12	238.32	7.24	0.50
Nitrogen intake (DM basis)	34.81	34.71	0.06	0.46
Fecal nitrogen	7.95	8.11	0.30	0.78
Urinary nitrogen	10.81	8.24	0.49	0.008*
TNO	18.76	16.36	0.59	0.042*
NAB	26.86	25.60	0.31	0.041*
NR	16.05	17.35	0.58	0.26
Nitrogen excretion of % intake	53.90	48.52	1.70	0.11
NPU %	46.09	51.47	1.70	0.11
BVFP %	58.94	67.67	1.94	0.02*
Nitrogen digestibility %	77.16	75.93	0.88	0.48
CTTAD	0.77	0.76	0.08	0.48
CAM	0.46	0.51	0.017	0.11
Plasma parameter				
Nitrogen g·mL^−1^	89.16	89.00	0.82	0.92
PUN mg·dL^−1^	10.3	10.9	0.27	0.27
Creatinine mg·dL^−1^ **	1.11	1.00	0.02	0.03 *
PUN:creatinine	7.62	8.88	0.23	0.006 **

* *p* < 0.0001 highly significant difference; *p* < 0.05 significant difference; *p* < 0.10 tendency of influence; *p* > 0.10 not significant; N = nitrogen. **Average daily gain (ADG); total nitrogen output (TNO); nitrogen absorption (NAB), retained nitrogen (NR); biological value of feed protein (BVFP), net protein utilization (NPU); coefficient of total tract apparent digestibility (CTTAD); coefficient of metabolizability (CAM).

**Table 3:** Effect of dietary extruded linseed:walnut meal 50:50 (*wt/wt*) mix on growth performance.

Specification * (kg)	SM Diet	LEW Diet	SEM	*p* Value
Initial weight	32.4	30.6	0.60	0.14
Final weight	55.64	54.68	0.97	0.62
BWG	23.10	24.10	0.26	0.06
ADG	1.10	1.14	0.03	0.053
Gain to feed	0.40	0.42	0.009	0.29
RFI	+1.08	+1.07	0.05	0.30

* BWG, body weight gain; ADG, average daily gain; RFI, residual feed intake.

**Table 4:** Effect of dietary extruded linseed:walnut meal 50:50 *(wt/wt)* mix on chemical composition in longissimus dorsi (LD) and semitendinosus (ST) muscles, heart, spleen, liver and cecum.

Specification (g × 100 g DM^−1^) *	SM Diet					LEW Diet					SEM	*p* Value **		
	LD	ST	Heart	Spleen	Liver	LD	ST	Heart	Spleen	Liver		Diet Effect	Tissue Effect	Diet × Tissue
DM %	26.41	25.12	19.49	27.12	25.36	25.56	28.03	18.24	23.09	28.29	0.89	0.97	0.009	0.47
EE	3.82	5.06	2.25	5.08	2.19	3.67	5.62	1.96	5.74	2.64	0.36	0.74	<0.0001	0.93
N	3.34	3.0	2.46	2.50	2.85	3.27	3.31	2.32	2.32	3.33	0.10	0.7	<0.0001	0.37

* DM, dry matter; CP, crude protein; EE, ether extract; N, nitrogen. ** *p* < 0.0001 highly significant difference; *p* < 0.05 significant difference, *p* < 0.10 tendency of influence; *p* > 0.10 not significant.

**Table 5:** Effect of dietary extruded linseed:walnut meal 50:50 (*wt/wt*) mix on centesimal fatty acid composition in longissimus dorsi (LD) and semitendinosus (ST) muscles, heart, spleen, liver and cecum.

Fatty Acids(%)*	SM	LEW	SM Diet						LEW Diet						SEM	*p* Value **		
	DIET		LD	ST	Heart	Spleen	Liver	Cecum	LD	ST	Heart	Spleen	Liver	Cecum		Diet Effect	Tissue Effect	Diet × Tissue
LA	16.42	1.6	13.05	13.9	23.19	11.49	20.19	16.71	13.18	14.7	22.47	10.84	21.31	13.84	0.67	0.82	<0.0001	0.63
ALA	0.5	1.83	1.35	1.36	0.43	1.00	0.38	0.46	1.55	1.44	2.34	1.69	1.86	2.79	0.12	<0.001	0.45	0.018
DPA	0.25	0.12	0.10	0.06	0.30	0.17	1.49	0	0.2	0.005	0	0.28	0.80	0.13	0.12	0.69	<0.0001	0.45
DHA	0.10	0.80	0	0	0.08	0	0.55	0	0.08	0.07	0,56	0.43	2,87	0	0.15	0.09	<0.0001	0.005
⅀n-6	21.92	21.25	15.35	16.36	30.22	14.37	36.59	20.10	15.21	16.81	27.34	14.24	36.21	16.69	1.37	0.85	<0.0001	0.17
⅀ n-3	1.92	3.58	1.98	1.75	1.36	1.96	3.31	0.69	2.32	1.83	3.38	3.19	6.67	3.21	0.27	0.005	<0.0001	0.004
n-6:n:3	15.03	6.52	7.73	9.35	22.13	10.03	11.49	29.46	6.63	9.30	8.12	4.49	5.42	5.17	1.12	0.004	0.012	0.019

* Fatty acids are expressed as % of total fatty acid methyl esters, FAME; LA, linoleic acid; ALA, α-linolenic acid; EPA, eicosapentaenoic acid; DPA, docosapentaenoic acid; DHA, docosahexaenoic acid. ** *p* < 0.0001 highly significant difference; *p* < 0.05 significant difference, *p* < 0.10 tendency of influence; *p* > 0.10 not significant.

**Table 6:** Pearson correlation between dietary and tissue n-3:n-6 polyunsaturated fatty acid (PUFA) and α-linolenic fatty acid (ALA).

Items	Tissue			
	n-6:n-3		ALA	
	r	P	r	P
Diet:				
n-6:n-3 ALA	0.51 * ‒0.51*	0.02 0.02	‒0.65 ** +0.65	0.002 0.002

* Correlation is significant at the 0.05 level (2-tailed). ** Correlation is significant at the 0.01 level (2-tailed).

**Table 7:** Effect of dietary extruded linseed:walnut meal 50:50 (*wt/wt*) mix on amino acid concentration in different tissues (longissimus dorsi (LD) and semitendinosus (ST) muscles, heart, spleen, liver, cecum).

Amino Acids (g × 100 g DM^−1^)	SM Diet					LEW Diet					SEM	*p* Value *		
	LD	ST	Heart	Spleen	Liver	LD	ST	Heart	Spleen	Liver		Diet Effect	Tissue Effect	Diet × Tissue
Threonine	3.42	3.26	3.31	2.44	2.73	3.38	3.31	3.33	2.66	2.74	0.07	0.18	0.35	0.58
Lysine	5.21	5.02	4.85	3.62	3.28	5.10	4.92	6.97	3.95	3.41	0.3	0.25	0.008	0.51
Cysteine	0.60	0.48	0.84	0.71	0.75	0.69	0.67	0.86	0.66	0.83	0.08	0.01	0.034	0.03
Methionine	1.73	1.47	1.75	1.43	1.51	1.96	1.73	1.75	1.81	1.66	0.1	0.002	0.21	0.21
